# Acyl‐CoA synthetase expression in human skeletal muscle is reduced in obesity and insulin resistance

**DOI:** 10.14814/phy2.15817

**Published:** 2023-09-19

**Authors:** Margarete Poppelreuther, Anne‐Marie Lundsgaard, Pernille Mensberg, Kim Sjøberg, Tina Vilsbøll, Bente Kiens, Joachim Füllekrug

**Affiliations:** ^1^ Molecular Cell Biology Laboratory, Internal Medicine IV University of Heidelberg Heidelberg Germany; ^2^ Section of Molecular Physiology, Department of Nutrition, Exercise and Sports, Faculty of Science University of Copenhagen Copenhagen Denmark; ^3^ Clinical Research Steno Diabetes Center Copenhagen Herlev Denmark; ^4^ Department of Clinical Medicine, Faculty of Health and Medical Sciences University of Copenhagen Copenhagen Denmark

**Keywords:** ACSL1, fatty acyl‐CoA synthetase activity, human skeletal muscle, insulin resistance, type 2 diabetes

## Abstract

Upon intramuscular entry, fatty acids are converted to amphiphatic fatty acyl‐CoAs by action of the acyl‐CoA synthetase (ACS) enzymes. While it has been reported that insulin resistant skeletal muscle shows an accumulation of fatty acyl‐CoAs, the role of the enzymes which catalyze their synthesis is still sparsely studied in human muscle, in particular the influence of obesity, and insulin resistance. We analyzed muscle biopsies obtained from normal weight controls (*n* = 7, average BMI 24), males/females with obesity (*n* = 7, average BMI 31), and males/females with obesity and type 2 diabetes (T2D) (*n* = 7, average BMI 34), for relevant ACS (long‐chain acyl‐CoA synthetase 1 (ACSL1), −3 (ACSL3) and − 4 (ACSL4), fatty acid transport protein 1 (FATP1) and − 4 (FATP4)). The mRNA expression was determined by real‐time PCR, and total oleoyl‐CoA synthetase activity was measured. In the males/females with obesity and T2D, the response to 16 weeks of exercise training with minor weight loss was evaluated. ACSL1 is the dominantly expressed ACS isoform in human skeletal muscle. The content of total ACS mRNA, as well as ACSL1 mRNA, were lower in muscle of males/females with obesity and T2D. Exercise training in the males/females with obesity and T2D increased the total ACS enzyme activity, along with a lowering of the HOMA‐IR index. The capacity for synthesis of fatty acyl‐CoAs is lower in skeletal muscle of obese males/females with T2D. This suggests a decreased ability to convert fatty acids to fatty acyl‐CoAs, which in turn may affect their entry into storage or metabolic pathways in muscle. Thus, the accumulation of fatty acyl‐CoAs in the obese or insulin resistant state that has been shown in previous reports is not likely to result from increased fatty acid acylation. The upregulation of ACS activity by exercise training appears beneficial and occurred concomitantly with increased insulin sensitivity.

## 1 INTRODUCTION

Upon entry into the muscle cell, either by diffusion or via fatty acid transport proteins (FATP), fatty acids are trapped after a CoA group is bound by acyl‐CoA synthetase (ACS) (Fritzen et al., 2020). Fatty acyl‐CoAs are amphipathic molecules, and the acylation of the fatty acids enables further metabolic processing, and is required before oxidation and storage can occur (Digel et al., 2009). ACS are thus essential enzymes to cellular lipid metabolism, involved in fatty acid uptake (by direct or indirect regulation) and intracellular channeling of fatty acids. It is believed that the channeling of fatty acyl‐CoAs toward either β‐oxidation or esterification/lipid synthesis is determined by ACS isoform compartmentalization in the cell (Cooper et al., 2015).

Several isoforms of ACS and FATP exist and vary in their tissue distribution pattern, intracellular location, and affinity for fatty acids, suggesting that each isoform has a distinct function in channeling fatty acids into metabolic pathways. The 13 mammalian long chain ACS enzymes are grouped into three subfamilies based on their sequence similarities: the long‐chain (ACSL), very long‐chain ACSVL/FATP, and the bubblegum long chain ACS. Enzymes of all groups contribute to activation of the most abundant long chain fatty acids in the organism. Five ACS enzymes are previously found to be expressed at significant amounts in skeletal muscle: ACSL1, ACSL3, ACSL4, FATP1, and FATP4. The study of their subcellular localization has been methodological challenging. Also, the ACS move intracellularly, and their localization likely also depend on the cell type studied. Nevertheless, it appears that ACSL1 is present at endoplasmic reticulum (ER) and mitochondria (Milger et al., 2006), ACSL3 is present at ER and lipid droplets (Poppelreuther et al., 2012), ACSL4 may have more ubiquitous intracellular localization (Küch et al., 2014), FATP1 at mitochondria (Beever et al., 2020), and FATP4 at ER (Milger et al., 2006). In Figure 2 we illustrate the proposed localizations of this five ACS enzymes. The ACS enzymes are found to increase sarcolemmal fatty acid uptake despite their intracellular localization, likely via metabolic trapping of the fatty acids (Milger et al., 2006; Zhan et al., 2012).

If fatty acid uptake exceeds the capacity for channeling toward lipid synthesis or β‐oxidation, fatty acyl‐CoA will accumulate in the cell, either in the cytosol or in mitochondria. To this end, it has been shown by overexpression studies in liver cells and rodent liver that the intracellular fatty acyl‐CoA content is subject to regulation by the ACS protein content, as shown for ACSL1 (Parkes et al., 2006).

An association between skeletal muscle fatty acyl‐CoA content and obesity and insulin resistance has been suggested. Hence, 90% higher content of fatty acyl‐CoAs has been obtained in skeletal muscle of obese compared with lean women (Hulver et al., 2003), and a negative correlation between whole‐body insulin sensitivity and muscle fatty acyl‐CoA content were obtained in men with a wide range in BMI (Ellis et al., 2000). Finally, Acipimox inhibition of endogenous fatty acid availability reduced skeletal muscle fatty acyl‐CoA by 25%, concomitantly with increased whole‐body insulin sensitivity in males/females with T2D (Bajaj et al., 2005). The intriguing question is whether fatty acyl‐CoA accumulation result from increased ACS enzyme action or reduced entry into metabolic and neutral storage pathways.

The aim of this study was to measure the expression of different ACS isoforms and total ACS activity in human skeletal muscle, while investigating the regulation by obesity and insulin resistance (T2D). Moreover, the effect of exercise training on ACS activity was investigated in males/females with T2D.

## 2 MATERIALS AND METHODS

### 2.1 Subject characteristics

Three groups of volunteers were included: normal weight healthy males/females (lean) (*n* = 7) males/females with obesity and normoglycemia (obese) (*n* = 7) and obese males/females with T2D (obese T2D) (*n* = 7). Participant characteristics are listed in Table 1. The obese males/females, and the obese males/females with T2D represent a sub‐cohort from two previous studies (Fritzen et al., 2015; Mensberg et al., 2017). Signed informed consent was achieved from each individual after oral and written information prior to inclusion. The studies were performed in accordance with the Declaration of Helsinki II and approved by the Copenhagen Ethics Committee (KF 01261127, H‐3‐2010‐058, and H‐4‐2011‐073) and the Ethics Committee of the Heidelberg Medical Faculty (S‐432/2012).

**TABLE 1:** Characteristics of the three participant groups: lean males/females, males/females (lean), obese males/females, males/females without diabetes (obese) and obese males/females, males/females with T2D (obese T2D). Data are means −/+SD.

	Lean	Obese	Obese T2D
Female/male	6/1	5/2	4/3
Age, y	26 ± 3	40 ± 13	54 ± 13
Body weight, kg	84.3 ± 7.6	93.9 ± 8.9	100.4 ± 13.8
BMI, kg·m‐^2^	23.8 ± 1.7	30.5 ± 3.3	34.0 ± 5.4
VO_2_max, l·min^−1^	4.4 ± 0.4	2.7 ± 0.3	2.6 ± 0.7
VO_2_max, l·kg^−1^·min^−1^	51.6 ± 2.5	28.4 ± 2.3	25.2 ± 5.8

Abbreviations: BMI, body mass index; VO_2_max, maximum oxygen uptake.

### 2.2 Sampling protocol for muscle biopsies

All participants arrived in the morning after an overnight fast (10 h). After 30 min of rest, a biopsy was obtained from the vastus lateralis muscle by a modified Bergström needle with suction under local anesthesia. Biopsies were carefully rinsed in ice‐cold saline, and immediately frozen in liquid nitrogen. Before the analyses, biopsies were freeze‐dried and dissected free for connective tissue, blood, and fat under microscope. Post‐training muscle biopsies of the males/females with T2D were obtained in the fasting state 42–75 h after the last training exercise bout.

### 2.3 Exercise training intervention and blood sampling in males/females with T2D

The males/females with T2D underwent 16 weeks of supervised exercise training, consisting of three supervised 60‐min training sessions per week (two indoor cycling sessions at average intensity of 65% to 85% of maximum heart rate, and one session consisting of eight different resistance exercises) (Mensberg et al., 2017). Before and after the training intervention, a fasting venous blood sample from an antecubital vein was obtained, concomitant with the muscle biopsies. On a separate day, the physical fitness level (VO_2_max) was estimated from a two‐step sub‐maximal bicycle test at pre‐ and post‐intervention.

### 2.4 Plasma analyses

Plasma glucose was determined with an ABL615 analyzer (Radiometer Medical), and plasma insulin was measured by ELISA (ALPCO).

### 2.5 Expression analysis by quantitative real‐time PCR (qPCR)

Total RNA was prepared from 7 to 10 mg of human muscle tissue with the ReliaPrep™ RNA Tissue Miniprep System (#Z6110, Promega, Madison, WI). Random priming with hexanucleotides was used for reverse transcription (SuperScript® III First‐Strand Synthesis SuperMix, (#18080–400, Invitrogen, Carlsbad, CA). Levels of ACSL1 (NM_001995.3), ACSL3 (NM_004457.3), ACSL4 (NM_001318510.1), FATP1 (NM_198580.3) and FATP4 (NM_005094.3) mRNA were determined by efficiency corrected relative quantification on an Applied Biosystems (Foster City, CA) 7500 Fast Real‐Time PCR System. Transcripts were detected with SYBR Green (Power SYBR Green Master Mix, #4367659, Applied Biosystems), and quantity was determined by comparison to a calibration curve obtained with five different dilutions of the corresponding plasmids (Poppelreuther et al., 2018). Normalization was relative to the quantity of ß‐actin transcripts (NM_001101.3), which were not different between groups. Real‐time PCR primers were verified to give a single product by agarose gel electrophoresis and melting curve analysis (for primer information see Table 2).

**TABLE 2:** Sequence information about the primers used for quantitative real‐time PCR analysis in 5′ to 3′ orientation.

Gene	Sense primer (5′ to 3′)	Antisense primer (5′ to 3′)
ACSL1	CTTCTGGTACGCCACGAGAC	GTCGCTGTCAAGTAGTGCG
ACSL3	GATGTTGGGTCAGAAACCAAAGA	ATGGCTGGACCTCCTAGAGTG
ACSL4	CTGGCCGACCTAAGGGAGT	ACATGAGCCAAAGGCAAGTAG
FATP1	GGACCCCAACGCGATATAC	GCCTCGTCTTCTGGATCTTG
F saiATP4	GTGATGTGCTGGTGATGGAC	CTCCAAGACCTGAGCAAAGC
ß‐Actin	GGACTTCGAGCAAGAGATGG	AGCACTGTGTTGGCGTACAG

### 2.6 Acyl‐CoA synthetase activity

Total cellular oleoyl‐CoA synthetase activity was determined by a radiometric assay (Füllekrug & Poppelreuther, 2016; Milger et al., 2006). For 30 min, 7–15 mg of muscle tissue was lysed on ice with 1% Triton X‐100, 130 mM KCl, 25 mM Tris–HCl, pH 7.4 and homogenized using a teflon pestle. Cell debris was sedimented by centrifugation at 4°C for 5 min at 10000 x g. 10 μL of the supernatant was incubated for 10 min at 30°C in 100 mM Tris pH 7.4, 5 mM MgCl_2_, 200 μM dithiothreitol, 10 mM ATP, 0.2% Triton X‐100, 20 μM [^14^C]‐oleate (specific activity 10 Ci/moL, supplied by NEC #661) and 200 μM CoA. The incubation was terminated by the addition of Dole's solution (isopropanol‐n‐heptane‐H_2_SO_4_, 40:10:1) and unreacted oleate was extracted four times with n‐heptane. The remaining oleoyl‐CoA in the aqueous phase was quantified by scintillation counting with a Wallac 1414 liquid scintillation counter (Wallac Oy, Perkin Elmer).

### 2.7 Intramyocellular triacylglycerol

For determination of IMTG, 400 μL tetraethylammoniumhydroxide was added to 2 mg freeze‐dried muscle fibers in a glass tube with a screw cap. After incubation overnight 175 μL 3 mol perchloric acid/L was added and the sample was centrifuged at room temperature for 10 min at 3000 x g. The supernate was neutralized with 250 μL 2 mol KHCO_3_/L and the glycerol concentration was measured fluorometrically.

### 2.8 Statistical methods and charts

**FIGURE 1:**
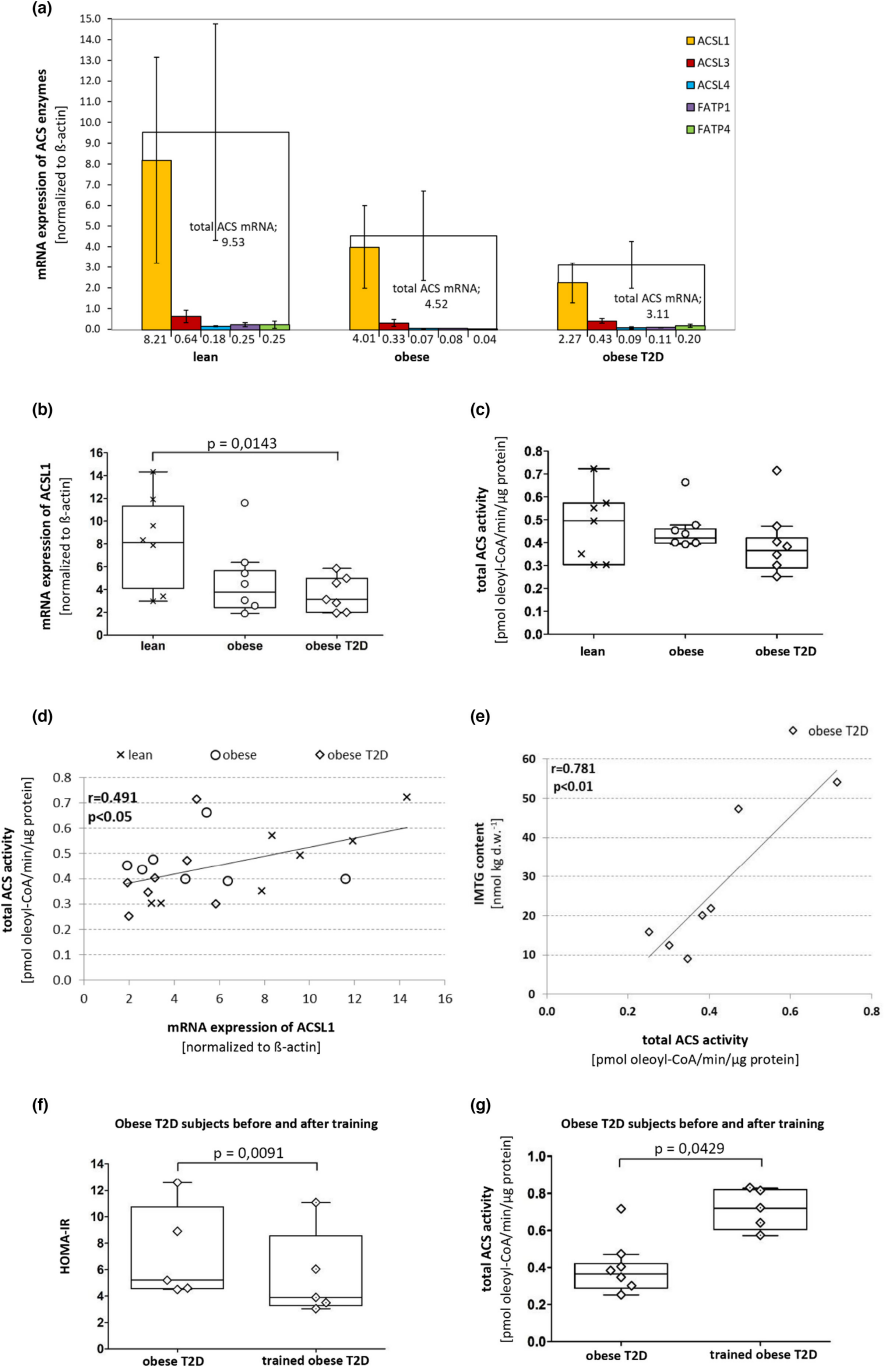
ACSL and ACSVL/FATP expression and total ACS activity. (a) Skeletal muscle mRNA content of the five long‐ (ACSL) and very long‐ (ACSVL/FATP) chain acyl‐CoA synthetase (ACS) enzymes in lean, obese, and obese males/females with T2D. (b) ACSL1 mRNA expression. (c) total acyl‐CoA synthetase (ACS) activity. (d) Correlation between total ACS activity and ACSL1 mRNA expression (Pearson correlation coefficient *r* = 0.49; *p* < 0.05). (e) In the obese males/females with T2D, correlation between total ACS activity and intramyocellular triacylglycerol (IMTG) content (Pearson correlation coefficient *r* = 0.78; *p* < 0.01). Before and after 16 weeks of exercise training, HOMA‐IR index was obtained (f) and skeletal muscle ACS activity measured (g). *n* = 7 in each group. *n* = 5 obese males/females with T2D before and after training. * *p* < 0.05 compared with lean. # *p* < 0.05 compared with pretraining. Data are means±SD for mRNA content (a), for data in B + C and F + G, box plots illustrate the median and quartiles of the dataset, with individual data points. In B + C, data were compared with the lean group using an unpaired Student's t‐test. Pearson's correlational analysis was applied to test associations between data in D + E. In F+ G Paired Student's *t*‐test was applied for comparison of males/females with T2D before and after exercise training. Measurements of ACS enzyme activities (c), (d), (e) and (g) were normalized to the total ACS activity measured, in the same experiment, in a standardized COS cell pellet.

For participant characteristics, and mRNA data in Figure 1a, data are means ± SD. Boxplots were generated using Prism 5, 5.01 (GraphPad software), graphically depicting the median and quartiles of the dataset (data in Figure 1b +C, 1F + G). All statistical analyses were performed using SPSS statistics 24.0 (IBM). Data was tested for equality of the variances by using Levene's Test. Sample size calculations on the main outcomes; ACSL activity and—mRNA, were not done prior to the analysis. Differences the obese males/females and obese males/females with T2D compared with normal weight were analyzed using an unpaired two‐tailed Student's *t*‐test. Paired Student's *t*‐test was applied for comparison of males/females with T2D before and after supervised exercise training. Pearson's correlational analysis was applied to test associations between data. A *p*‐value <0.05 was considered to indicate statistical significance. The applied statistics are indicated in the figure legend.

## 3 RESULTS

### 3.1 Lower transcription of total ACS enzymes and ACSL1 in insulin resistant skeletal muscle

The body mass index (BMI) of the lean, obese males/females and the obese males/females with T2D are shown in Table 1. Importantly, BMI was similar in the two groups with obesity (Table 1). The obese group had a normoglycemic homeostatic model assessment of insulin resistance (HOMA‐IR) index of 1.6 ± 0.5 index values, while HOMA‐IR amounted to 7.2 ± 3.2 index values in the group with T2D, revealing insulin resistance (*p* < 0.01).

The mRNA expression analysis revealed that total ACS mRNA content in muscle of males/females with obesity and T2D comprised 47% and 33%, respectively of the total content in lean males/females (Figure 1a). Of the measured mRNA abundance of the ACS isoforms, ACSL1 showed the greatest mRNA expression in all three groups. ACSL1 mRNA content in the obese and obese T2D group comprised 63% (*p* = 0.13) and 45% (*p* < 0.05), respectively, of the ACSL1 mRNA content in the lean males/females (Figure 1b).

### 3.2 Total ACS enzyme activity corresponds to ACSL1 mRNA expression

For total ACS activity in muscle, no significant differences were obtained between the lean, obese and obese T2D group (Figure 1c), with a numerically 13% lower ACS activity in muscle of males/females with obesity and T2D compared with lean males/females. A significant correlation was, however, evident between the mRNA expression of ACSL1 and ACS activity for all three groups (Figure 1d, *r* = 0.49, *p* < 0.05). In skeletal muscle of the males/females with T2D, the basal resting IMTG content was determined and the association to total ACS activity investigated. Interestingly, total ACS activity showed a strong correlation with intramyocellular triacylglycerol (IMTG) content in muscle (*r* = 0.781, *p* < 0.01) (Figure 1e), supporting a role for the ACS enzymes in neutral lipid storage in human muscle.

### 3.3 Endurance training increases total ACS activity in males/females with obesity and T2D

A subset of the males/females underwent 16 weeks of supervised exercise training. The exercise training increased maximal oxygen uptake (VO_2_peak) by 29% (2.4 ± 0.4 L/min to 3.1 ± 0.5 L/min, *p* < 0.05), while body weight loss was moderate (4.1 ± 1.7 kg, p = 0.08). The training intervention lowered HOMA‐IR from 7.2 ± 1.5 to 5.5 ± 1.5 index values (Figure 1f). In skeletal muscle, exercise training increased ACS activity by 74% in muscle of males/females with T2D compared with pretraining (*p* < 0.05) (Figure 1g). At post‐training, the ACS activity level even exceeded that measured in the lean control group (0.72 ± 0.05 versus 0.47 ± 0.06 pmol/min/ug protein in the lean).

## 4 DISCUSSION

The results of this study pinpoint ACSL1 as the most prominent transcribed ACS enzyme in human skeletal muscle, independent of the presence of obesity or insulin resistance. Other ACSL or ACSVL isoforms (ACSL3, ACSL4, FATP1, and FATP4) were also transcribed, but their proportion was lower, as apparent for all three groups. Hence, determinated from the mRNA abundance, ACSL1 is likely contributing the majority of total ACS activity in human skeletal muscle. This is consistent with data in mice where muscle‐specific knockdown of ACSL1 results in 91% reduction of total ACS activity (Li et al., 2015). Other factors, such as compartmentalization and the affinity to fatty acid substrates may also play a role for the relative importance of ACSL1 in determining total ACS activity.

**FIGURE 2:**
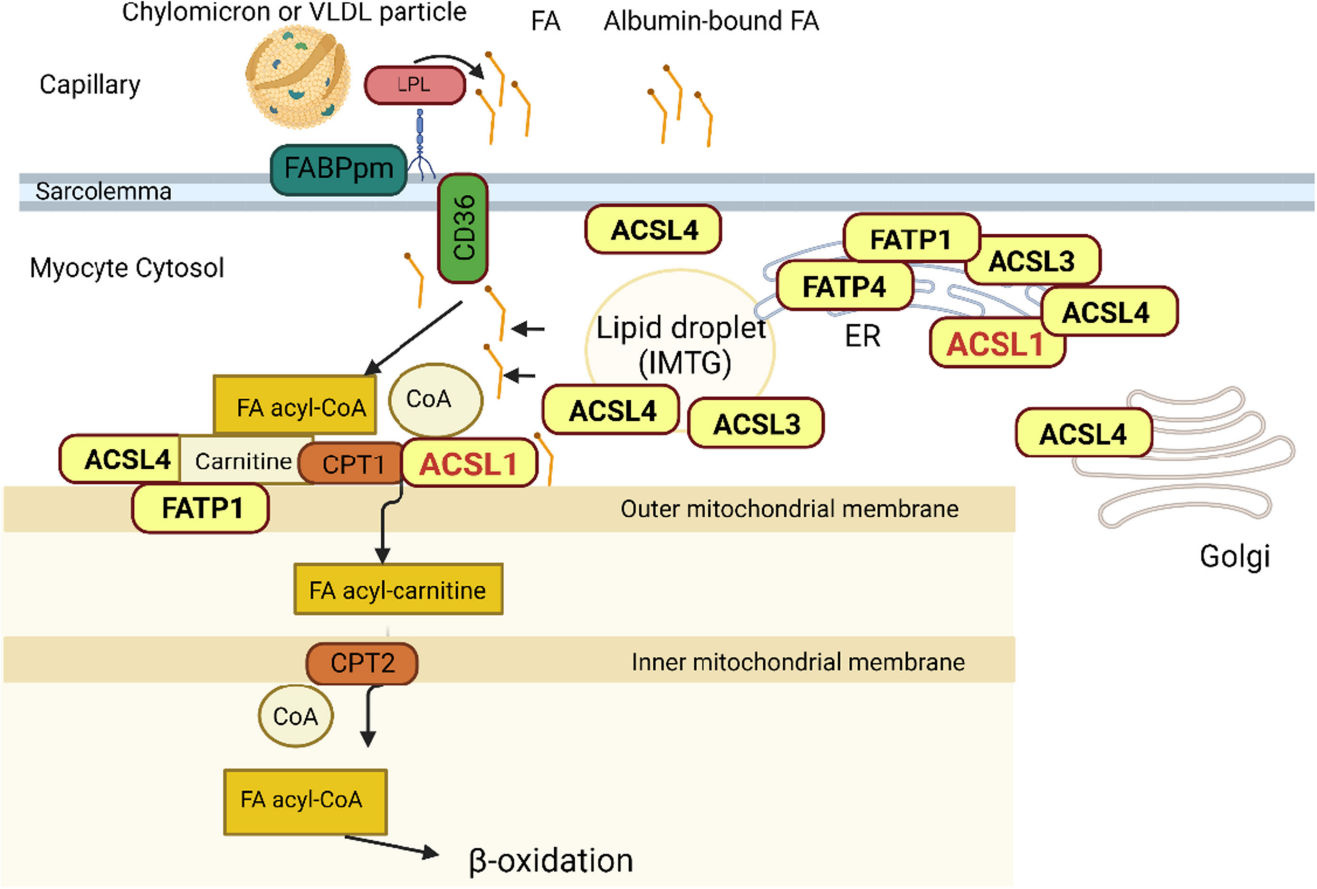
ACS enzymes and their role in lipid metabolism in skeletal muscle. Illustration of the proposed localization of the different ACS enzymes, based on the available evidence. ACSL, long‐chain acyl‐CoA synthetase, FA, fatty acid, FATP, fatty acid transport protein. The localization of the ACS enzymes must be considered with caution, as uncertainties exists (likely related to different tissues being investigated and the use of different methods (i.e., histological, fractionation, or others). The enzymes may therefore also be present at other cellular compartments.

From studies in hepatocytes and C2C12 myotubes, ACSL1 is reported to be located at the outer mitochondria membrane, building a fatty acid transfer complex with carnitine palmitoyl transferase 1 (CPT1) (Distler et al., 2007; Nan et al., 2021) (illustrated in Figure 2) and the voltage‐dependent anion channel (VDAC) (Lee et al., 2011) Hence, muscle‐specific ACSL1 knockout in mice led to compromised fatty acid oxidation (Li et al., 2015; Zhao et al., 2019).

ACSL1 also localizes to the ER (illustrated in Figure 2), and though the fate of acyl‐CoA produced by ER localized ACSL1 is not clearly understood, this site is likely involved in lipid synthesis, and it has been shown in adipocytes that siRNA silencing of ACSL1 led to reduced TG accumulation (Joseph et al., 2015).

ACSL1 mRNA content, and the total ACS mRNA content (ACSL1, ACSL3, ACSL4, FATP1, FATP4), were lower in muscle of healthy obese and obese insulin resistant males/females compared with normal weight controls. Concomitantly, we found that the maximal ACS activity was similar between the obese and obese insulin resistant compared with normal weight males/females. In regard to this discrepancy, we have previously found in C2C12 muscle cells that the intrinsic enzyme activity (of FATP4/ACSVL4) could be regulated independently of expression level (Digel et al., 2011).

We could not demonstrate increased ACS gene expression or increased ACS activity in the obese insulin resistant muscle samples, which suggests that the fatty acid acylation capacity is not upregulated in this state (but rather downregulated, determined from the mRNA abundance). This suggests that a regulatory link between the activity of the ACS enzymes and muscle insulin resistance (due to a potential accumulation of fatty acyl‐CoAs or other lipid intermediates) is missing. To this end, fatty acyl‐CoA content was shown to be identical between obese males/females with T2D, normal weight and endurance trained males/females (Bruce et al., 2003), which suggest that neither fatty acyl‐CoA content nor ACS expression seems to be culprit in muscle insulin resistance. We did not have sample material to measure fatty acyl‐CoA content in muscle of the insulin resistant group. However, IMTG was determined and a correlation between IMTG storage and ACS activity was obtained in the obesity and T2D group pointing that ACS is important for fatty acid esterification into neutral lipids. In support, suppression of ACSL1 in human primary hepatocytes blunted FA storage as TG (Li et al., 2020).

The lower ACS mRNA content in the obese and obese T2D group could potentially relate to a lower oxidative capacity (as indicated by the low VO_2_max values of 28 and 25 mL/kg/min) and hence a lower ß‐oxidation rate. It is also conceivable that a potential excess of fatty acyl‐CoA levels in the muscle of males/females with obesity or T2D could lead to feedback inhibition of the ACS, either directly or by regulating the activity of transcription factors (Faergeman & Knudsen, 1997), such as PPARα and –γ (Martin et al., 1997). Finally, the expression of ACSL1 has been shown to be regulated by insulin signaling in hepatocytes (Ning et al., 2011). Hence, the observed downregulation of ACSL1 expression could result from compromised insulin signaling in muscle of males/females with T2D.

Skeletal muscle ACS activity was upregulated by 74% following aerobic exercise training in the obese males/females with type 2 diabetes compared with before, concomitantly with improved whole‐body insulin sensitivity, documented by the lowering of HOMA‐IR. Suggesting that exercise itself played the major regulatory role, the training period was only associated with minor weight loss, and participants maintained their habitual diet and physical activity (Mensberg et al., 2017). We have previously obtained an increase of skeletal muscle ACSL1 protein abundance at the single‐fiber level in normal weight men in response to 12 weeks endurance training (Deshmukh et al., 2021). This could support that upregulation of ACSL1 protein may be the ACS isoform responsible for the exercise training‐induced increased ACS activity. One previous study demonstrated an exercise training‐induced increase in skeletal muscle ACSL1 mRNA along with increased postprandial fatty acid oxidation assessed by tracers in sedentary normal weight men (Bergouignan et al., 2013), pointing to a potential role of ACSL1 in increased fatty acid oxidation capacity. Limitations of the present study include the low number of participants in each group. Moreover, the participants with T2D and obesity were older than the lean participants. Each group included male and female participants, and while we did not obtain sex‐specific regulation of ACSL activity or mRNA, this needs to be confirmed in a higher number of samples. Finally, analysis of fatty acyl‐CoA content was not possible due to a limited amount of muscle sample.

Taking together, the present data suggest that ACSL1 is downregulated in insulin resistant skeletal muscle, but is a relevant target upregulated by exercise training, which has potential to increase the utilization of fatty acids through ß‐oxidation.

## CONFLICT OF INTEREST STATEMENT

All authors declare that the study was conducted without financial or conflicts of interest. T.V. has served on scientific advisory panels, been part of speaker's bureaus, and served as a consultant to and/or received research support from Amgen, AstraZeneca, Boehringer Ingelheim, Eli Lilly, Gilead, GSK, Mundipharma, MSD/Merck, Novo Nordisk, Sanofi, and Sun Pharmaceuticals.
